# Preeclampsia among Omani women: prevalence and associated risk factors

**DOI:** 10.3389/fgwh.2026.1811134

**Published:** 2026-07-15

**Authors:** Lilian Birungi, Vidya Seshan, Blessy Prabha Valsaraj, Jansi Rani Natarajan, Najla Majid Al Battashi, Azza Nasser Al Jabal, Ream Langhe

**Affiliations:** 1 College of Nursing, Sultan Qaboos University, Muscat, Oman; 2 Department of Nursing College of Health Sciences, University of Sharjah, Sharjah, United Arab Emirates; 3 College of Health Sciences, University of Buraimi, Al Buraimi, Oman; 4 Maternity Unit, Royal Hospital, Muscat, Oman; 5 Department of Clinical Science, College of Medicine, University of Sharjah, Sharjah, United Arab Emirates

**Keywords:** hypertension, Oman, preeclampsia (PE), pregnancy, prevalence, risk factors

## Abstract

**Background:**

Preeclampsia is a significant pregnancy complication characterized by high blood pressure and potential organ damage, posing substantial risks to both maternal and neonatal health.

**Objectives:**

This study aimed to identify the prevalence of Preeclampsia and associated risk factors among Omani women.

**Design:**

An unmatched retrospective case-control study.

**Methods:**

A case-control study was conducted at two tertiary hospitals in Oman. Data were collected retrospectively from 668 women (167 with PE and 501 normotensive controls), including demographic, obstetric, and clinical variables. Data was analyzed using Pearson Chi-square tests and independent t-tests for associations or differences. Binary logistic regression was used to explore independent predictors of Preeclampsia, excluding diagnostic criteria (hypertension, proteinuria) from the model.

**Results:**

The cohort-based prevalence of preeclampsia among the screened study population was 25.0%. Women with preeclampsia had significantly higher gravidity (*p* = 0.017) and parity (*p* < 0.001) compared with controls. In multivariate logistic regression analysis, lower gestational age at delivery (AOR =  0.92 per additional week; 95% CI: 0.87–0.97; *p* = 0.003) and higher gravidity (AOR=1.45 per unit increase; 95% CI: 1.05–2.01; *p* = 0.025) were identified as independent predictors of Preeclampsia.

**Conclusion:**

Gestational age and gravidity are significant predictors of PE in Omani women, providing crucial baseline data for national maternal health strategies and highlighting the need for increased prenatal attention for women with shorter gestations and higher gravidity.

## Introduction

Preeclampsia (PE), defined by the American College of Obstetrics and Gynecology (ACOG, 2020) as hypertension after 20 weeks of gestation with proteinuria in previously normotensive women (1). PE is a disorder associated with high rates of maternal and neonatal mortality, necessitating prompt management. In Oman, the definition and treatment of PE closely resemble the international guidelines, such as the European Society of Hypertension (ESH) and the American College of Obstetricians and Gynecologists (ACOG), concerning diagnostic criteria, such as the presence or absence of proteinuria along with end-organ dysfunction, the onset of hypertension after 20 weeks of gestation, and a blood pressure of ≥140/90 mmHg on two occasions (2–4). The use of magnesium sulfate for seizure prevention, antihypertensives (labetalol, methyldopa, and nifedipine), and customized delivery timing are all examples of management practices in Oman that are in line with international guidelines, albeit modified to meet the needs of the local healthcare system and referral procedures (4).

Literature shows that the common maternal risk factors linked to a higher incidence and recurrence of PE include primigravity, obesity, advanced maternal age (≥35 years), and a family history of PE (5). PE has also been closely associated with elevated body mass index due to excessive gestational weight gain and gestational diabetes mellitus (6). The pathophysiology of PE involves several interconnected systems and remains complex; it includes abnormal placentation and impaired angiogenesis. Placental dysfunction due to aberrant placentation and angiogenesis, also plays significant roles in PE (7). However, there is conflicting evidence regarding risk reduction with increasing parity, even though primigravid women are at a much higher risk for PE (8).

Early detection of maternal risk factors for PE serves as a crucial guide for prompt diagnosis, management, and appropriate referrals of severe cases to tertiary healthcare facilities, thereby reducing adverse effects on maternal and fetal health. The impact of PE on maternal and fetal outcomes is significant. For instance, globally, the prevalence of PE varies from 0.2% to 16.7% in Asia, 2.8% to 5.2% in Europe, and up to 62.2% in some regions of Africa (9). There are greater rates of PE recorded in low-income countries because of a lack of maternal health services (10). Iran and Saudi Arabia have a rate of 0.05% and 5.37%, respectively (11).

PE is the most prevalent hypertensive condition during pregnancy in the Gulf region (12). This seems to be a global phenomenon, because countries including the USA, China, and Malaysia have also shown notable increases in the number of severe cases (13). Accordingly, the (14), ICD-11 divides PE into four categories: mild to moderate (JA24.0), severe (JA24.1), HELLP syndrome (JA24.2), and unspecified types (JA24.Z). PE can also be classified as early-onset (less than 34 weeks) or late-onset (more than 34 weeks) (15). Early-onset PE is better predicted by biomarkers (16). Although blood pressure, proteinuria, and clinical symptoms are commonly used to determine the severity of PE, these standards can be arbitrary (17).

The current study aimed to investigate the prevalence and risk factors associated with PE among Omani women. PE was defined as proteinuria (≥1+) and hypertension (SBP ≥140 mmHg and/or DBP ≥90 mmHg) after 20 weeks of pregnancy, including those with a history of PE. The prevalence of PE was estimated as the ratio of PE cases to all births during 2021–2023 that were included in the study cohort. And the risk factors included factors such as cesarean delivery, BMI, parity, previous PE, gestational diabetes, and maternal age were among the risk factors evaluated.

## Methods

### Study design

An unmatched retrospective case-control design was used to evaluate the prevalence of PE and associated risk in women who gave birth at Royal Hospital (RH) and Sultan Qaboos University Hospital (SQUH). This design was ideal since it allowed for effective data retrieval over a predetermined time period and the investigation of correlations between variables (18, 19). The retrospective case-control approach is suitable for investigating conditions that have already occurred and for identifying associated risk factors from past exposures.

### Study setting

The study was carried out at two tertiary referral hospitals in Muscat, Oman. The two hospitals are government-owned and operated, providing direct healthcare services (20). The facilities are important referral hubs for high-risk pregnancies nationwide and provide comprehensive maternity and newborn care services. These hospitals were chosen due to their provision of secondary and tertiary healthcare services to individuals from diverse socioeconomic backgrounds, including both Omani and non-Omani nationals. Women who gave birth at the two facilities between January 2021 and December 2023 were studied. The study's controls were normotensive women who gave birth in the same hospitals throughout the same time period, and cases were women with a PE diagnosis after 20 weeks of pregnancy.

### Study population and participants

**Figure 1:**
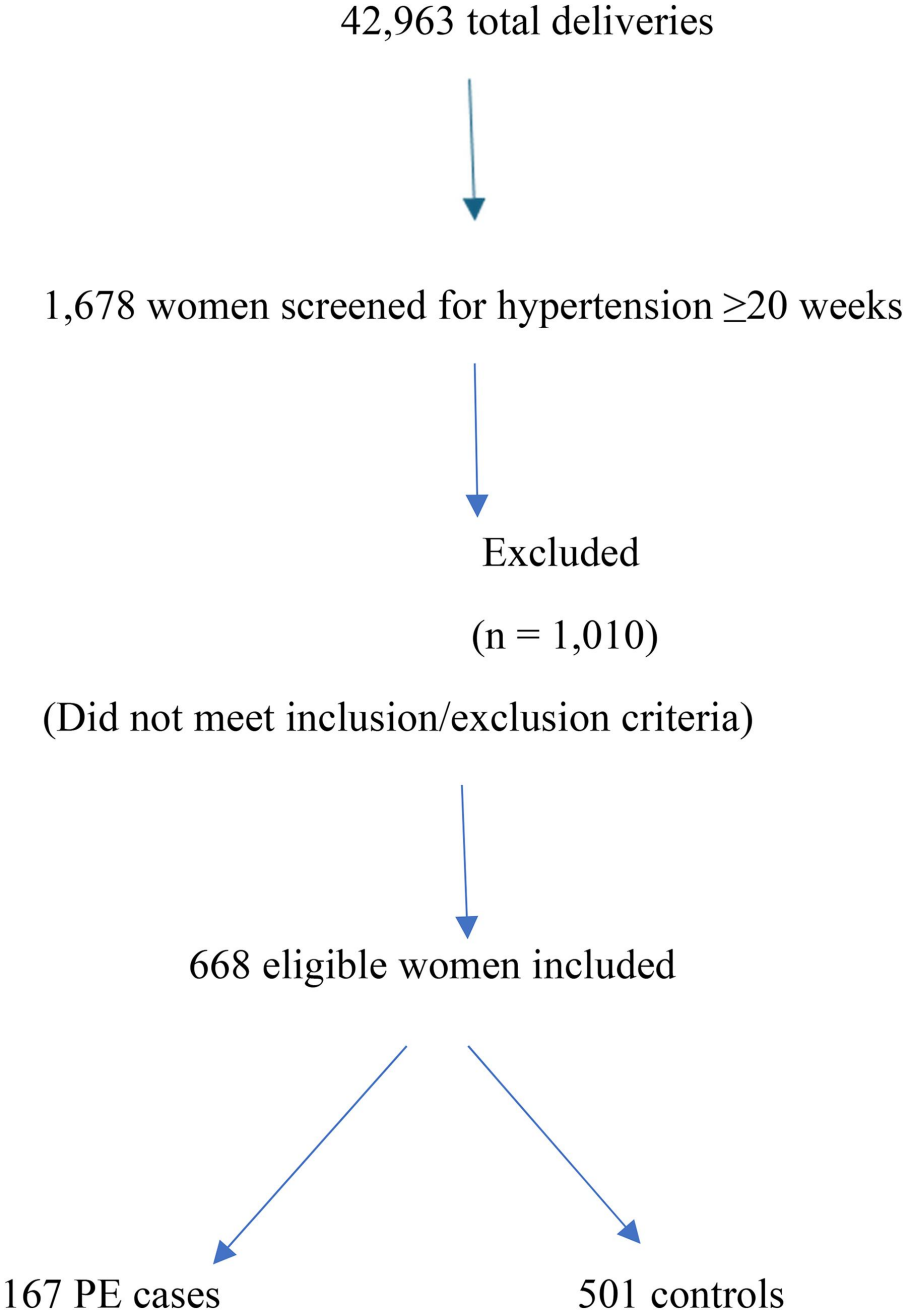
STROBE participant flow diagram.

The study focused on mothers who delivered their babies from January 1, 2021, to December 31, 2023, at SQUH and RH. The participants were identified using the Hospital's electronic medical tracking systems. The above timeframe was chosen to guarantee data consistency after the COVID-19 pandemic in 2020, which had disrupted healthcare systems with fewer hospital visits and fewer resources. Using hospital electronic data, preeclamptic women were discovered, and manual evaluation of medical files confirmed the diagnoses. Following screening based on predetermined inclusion and exclusion criteria, eligible participants were divided into two groups: cases, were women diagnosed with preeclampsia after 20 weeks of gestation based on documented (SBP ≥ 140 mmHg and/or DBP ≥ 90 mmHg) with proteinuria and/or clinical evidence of end-organ involvement, according to hospital diagnostic criteria aligned with ACOG and neonatal guidelines and controls were selected from women delivering during the same study period and hospitals who remined normotensive throughout pregnancy and had no documented diagnosis of hypertensive disorders of pregnancy. During the study period, 42,963 deliveries were recorded across the two hospitals. Among these, 1,678 singleton pregnancies with documented hypertension after 20 weeks of gestation were initially screened through the hospital electronic medical record systems. Medical records were subsequently reviewed manually to confirm eligibility according to the predefined inclusion and exclusion criteria. Of the screened records, 668 eligible participants were included in the final analysis, comprising 167 women with preeclampsia and 501 normotensive controls selected in a 1:3 ratio. Systematic sampling was used separately for cases and controls within each hospital, using calculated sampling intervals and lottery selection for the first record (Figure 1).

### Inclusion criteria and participant selection

The study included singleton pregnancies of women aged 15 to 50 years, 20 weeks of gestation or more, with hypertension newly identified during the current pregnancy after 20 weeks of gestation and without documented chronic hypertension before pregnancy. Only those who had been booked cases were eligible. Cases with major fetal anomalies, those undergoing dialysis, known hypertensive individuals, or those with chronic or active diseases were excluded from the study.

Women with a previous history of preeclampsia were not excluded if the current pregnancy fulfilled the diagnostic criteria for preeclampsia. Previous history of PE was considered part of the obstetric background rather than an exclusion criterion.

Participants’ records were selected using a systematic sampling technique, where the sampling interval (K) for cases and controls was determined by dividing the total number of cases and controls (N) by the desired sample size (n) for each group, a calculation conducted separately for each hospital, and using the calculated K value, patients records were selected at every K^th^ interval for both cases and controls, with the first study subject records chosen by the lottery method. Data collection was conducted with one case to three controls; the analysis was done in an unmatched manner. This is also based on several preeclamptic studies, such as those conducted by (21) and (22), who conducted unmatched case-control studies.

### Sample and data collection procedure

The sample size was calculated using the method by (23). To ensure statistical power, the study included 167 cases of PE and 501 controls, selected based on a ratio of 1 case to 3 controls. The study followed the guidelines outlined in the Strengthening the Reporting of Observational Studies in Epidemiology (STROBE) statement (24). PE was the independent variable, with dependent variables including associated risk factors (maternal age, gravidity, parity, abortion, GDM during pregnancy, urine protein, systolic/ diastolic pressure, BMI, Hb admission, and platelet count at admission). These variables facilitated the examination of the relationship between PE and risk factors for pregnant women.

A data collection sheet based on Salim, Nour Eldin (25), was validated through a pilot study involving 10% of the sample, ensuring the reliability and feasibility of data collection across both hospitals. Continuous on-site checks and cross-referencing with medical records maintained data accuracy. The data collection sheet questionnaire covered demographic details, maternal assessments, and laboratory results. Data was collected from June 2023 to February 2024.

### Bias

Several measures were taken to reduce biases. All eligible PE patients and controls during the study period were included to guarantee representativeness and minimize selection bias. By employing pretested, standardized data abstraction tools and providing training to research assistants who were midwives employed by the chosen hospitals, information bias was reduced. Only information that was collected contemporaneously was used, and the accuracy of the data was checked against hospital records.

### Statistical analysis

Data was analyzed using SPSS version 23 (26). Continuous variables (e.g., maternal age, BMI, hemoglobin) were assessed for normality using the Shapiro–Wilk test, Q-Q plots, and histograms to guide the choice between parametric and nonparametric tests. For normally distributed data Maternal age, BMI, blood pressure, and systolic and diastolic pressure demonstrated approximate normal distribution and were therefore analyzed using independent samples t-tests. The Mann–Whitney U test was applied to skewed data, and medians and interquartile ranges were provided. When comparing categorical variables, the Chi-square or Fisher's exact test was used as appropriate. For every analysis, *α* = 0.05 was chosen as the significance level. A two-step process was employed to identify the predictors. Initially, unadjusted correlations between each independent variable and PE were evaluated using bivariate logistic regression. An initial multivariable logistic regression model comprised variables that were clinically relevant or had *p*-values ≤ 0.25 in bivariate analysis. To prevent circular logic, all regression models did not include the diagnostic criteria for PE (systolic/diastolic blood pressure and urine protein levels). Non-significant confounders (*p* > 0.05) were eliminated from the final model. Crude odds ratios (CORs) and adjusted odds ratios (AORs) with 95% confidence intervals (CIs) are used to display the results.

### Ethical considerations

This study involved human participants and was a retrospective review of de-identified medical records from women who delivered between January 2021 and December 2023. Ethical approval was obtained from the Research and Ethics Committees of the College of Nursing (CON/MSN/2023/8), the College of Medicine and Health Sciences (MREC#3083), and the Ministry of Health, Oman (MOH/CSR/22/26915). A waiver of informed consent was granted due to the retrospective and anonymized design. The study was conducted in accordance with the Declaration of Helsinki and national regulations. No potentially identifiable human images or data are included in this study.

## Results

Between January 2021 and December 2023, 42,963 women gave birth at two selected hospitals. A total of 1,678 singleton pregnancies were screened for hypertension after 20 weeks. Of these, 668 women satisfied the study's inclusion and exclusion criteria; 501 of them were normotensive controls, and 167 had a diagnosis of preeclampsia. This resulted in a prevalence of PE of 25.0% (167/668) within the studied cohort. The characteristics of the participants included in the study are presented in Table 1. The participants were distributed between Hospital 1 (61%) and Hospital 2 (38%). The average age was 31.7 ± 5.4years, and there was no significant age difference between cases (32.3 ± 5.7 years) and controls (31.5 ± 5.3 years) at *p* = 0.92. The greatest number of cases was in the Muscat region (68.3%) compared to controls (59.3%), *p* < 0.05.

**Table 1:** Demographic and obstetric risk factors of study participants.

Characteristics	Category	Case (*n* = 167)		Control (501)		Chi-square value	*p*-value
		Freq	Percent	Freq	Percent		
Hospital	Hospital 1	104	62.3%	308	61.5%	0.034	0.854
	Hospital 2	63	37.7%	193	38.5%		
Governorates	Al Batinah	32	19.2%	123	24.6%	8.034*	0.045
	Muscat	114	68.3%	297	59.3%		
	Al Sharqiyah	10	6.0%	58	11.6%		
	Others	11	6.6%	23	4.6%		
Gravidity	Primigravida	46	27.5%	88	17.6%	8.194*	0.017
	2–3 pregnancies	60	35.9%	219	43.7%		
	4 + pregnancies	61	36.5%	194	38.7%		
Parity	Primipara	56	33.5%	99	19.8%	18.113***	0.001
	Para 1	41	24.6%	131	26.1%		
	Para 2–3	43	25.7%	202	40.3%		
	4+ (Multipara)	27	16.2%	69	13.8%		
Abortion	No	115	68.9%	343	68.5%	0.009	0.923
	Yes	52	31.1%	158	31.5%		
GDM during pregnancy	No	111	66.5%	354	70.9%	1.145	0.285
	Yes	56	33.5%	146	29.1%		
Urine Protein	None	91	54.5%	482	96.2%	178.677***	0.001
	+1-+4	76	45.5%	19	3.8%		

* is *p* < 0.05 statistically significant. ** is *p* < 0.01 highly statistically significant. *** is *p* < 0.001 very highly statistically significant.

Significant associations were found between PE, gravidity (*p* < 0.05), and parity (*p* < 0.01). Primigravida (27.5%) and women with 2–3 pregnancies (35.9%) were more common among cases, while controls had higher proportions of women with 4 + pregnancies. Most preeclamptic women were either primipara (33.5%) or para 2–3 (25.7%), compared to controls (19.8% and 40.3%, respectively), indicating a higher risk with fewer pregnancies and lower parity. The urine protein levels were also significantly associated with PE (*p* < 0.01), with 45.5% of cases showing proteinuria (+1 to +4) versus 96.2% of controls that had no proteinuria. PE was found to be associated with gravidity, parity, and urine protein levels, emphasizing the need for careful monitoring of these factors in clinical settings.

## Maternal risk factors of study participants

The independent samples’ t-test was used to assess differences between pregnant women with and without PE. The results presented in Table 2 show that women with PE had considerably higher systolic and diastolic blood pressure (both *p* < 0.001) than controls, as expected by the diagnostic criteria, supporting the validity of the case-control classification. Furthermore, the mean body mass index of women with PE was considerably greater (*p* < 0.001).

**Table 2:** Maternal risk factors of study participants.

Variables	Category	n	Mean	SD	t-value	*p*-value
Systolic pressure at admission	Case	167	158.81	18.417	26.974	<0.001
	Control	501	118.34	10.514		
Diastolic pressure at admission	Case	167	96.60	12.019	24.580	<0.001
	Control	501	71.36	9.745		
Maternal age	Case	167	32.26	5.718	1.690	0.092
	Control	501	31.45	5.263		
Body mass index	Case	167	31.56	6.603	3.425	<0.001
	Control	501	29.56	6.493		
Hemoglobin at admission	Case	167	11.27	1.423	-0.162	0.872
	Control	501	11.29	1.245		
Platelet count at admission	Case	167	216.40	68.584	−1.444	0.149
	Control	501	225.25	68.670		

All continuous variables presented in Table 2 demonstrated approximate normal distribution based on Shapiro–Wilk testing and visual assessment of Q-Q plots and histograms. Urine protein levels and blood pressure values presented in Tables 1, 2 are reported solely to validate the case–control classification based on diagnostic criteria for preeclampsia and are not considered independent risk factors under investigation.

## Predictors of PE among Omani women

The predictors of PE were identified using binary logistic regression, eliminating definitional criteria. The original multivariable model contained variables that were clinically significant or had a *p*-value < 0.25 in bivariate analysis. Gravidity and gestational age at birth were found to be significant independent predictors in the final model (Table 3). Higher gravidity was similarly linked to higher risks of PE (AOR: 1.45; 95% CI: 1.05–2.01; *p* = 0.025), as was shorter gestational age (AOR: 0.92 per week increase; 95% CI: 0.87–0.97; *p* = 0.003).

**Table 3:** Adjusted Odd ratio for non-definitional factors associated with PE among women.

Variable	*P*-value (COR)	COR	95% C. I	*P* value	AOR	95% C. I
			(lower–upper)			(lower–upper)
Gestational age at delivery (weeks)^1^	0.004	0.96	0.93–0.98	0.003	0.92	0.87–0.97
Gravidity^1^	0.21	1.08	0.95–1.22	0.025	1.45	1.05–2.01
Parity^1^	0.392	0.93	0.78–1.10	0.442	0.88	0.63–1.22
Maternal age (years)^1^	0.502	1.02	0.97–1.07	0.506	1.02	0.97–1.07
BMI (kg/m^2^)^1^	0.622	1.02	0.95–1.08	0.178	1.03	0.99–1.07
History of GDM (Yes vs. No)	0.784	0.96	0.70–1.30	0.316	1.25	0.81–1.92
Hemoglobin at admission (g/dL)^1^	0.788	0.99	0.91–1.08	0.661	0.97	0.84–1.12
Platelet count at admission (x10^3^/µL)^1^	0.512	1.00	0.99–1.00	0.450	1.00	0.99–1.00

1 Continuous variables were analyzed without centering or normalization. Analyzed as a continuous variable. The Adjusted Odds Ratio (AOR) represents the change in odds associated with a one-unit increase in the corresponding variable. For categorical variables, the reference category was the absence of exposure or condition.

## Discussion

Despite extensive attention to PE in developed countries, limited research has been conducted in the Middle East to ascertain its prevalence and associated factors. No studies have specifically explored PE in Oman and its impact on maternal and neonatal health. The findings of the current study show that the prevalence of PE in Oman is 25.0% within the studied cohort. And the rates reported in Jordan (1.3%), Bahrain (1.95%), Qatar (2.3%), and Lebanon (2.84%), Saudi Arabia (5.37%), Iraq (4.79%), Egypt (19.1%), Thailand (9.1%), and some African countries such as Uganda (4.3%), and Ghana (5.6%) (27–36). But direct comparison with population level prevalence estimates from other countries should be interpreted cautiously, as the current study reports a cohort-based prevalence derived from a screened hospital population rather than a national population-based estimate. At the international level, the prevalence of PE varies widely, with rates in Asia, Europe, South America, Oceania, and North America reported at 0.2–16.7%, 2.8%–5.2%, 1.8%–7.7%, 2.8%–9.2%, and 2.6%–4.0%, respectively (9). While not a population prevalence, this rate within the screened cohort provides paramount clinical insights.

The prevalence of PE reported by the current study could be influenced by the methods used in the study. Methodological differences, including study design (retrospective vs. prospective), case-finding methods, study duration, and healthcare settings, influence reported prevalence rates. Moreover, multicenter studies tend to provide more representative data, as seen in Qatar and Ghana, whereas single-center studies like those in Egypt and Iraq may have limited generalizability (29, 32, 33, 36). The study period also impacts prevalence rates, with shorter studies potentially missing seasonal fluctuations and evolving healthcare practices. We recommend that future studies use longitudinal methods over several years to capture more comprehensive data and variations due to season and other natural events.

## The risk factors of PE among Omani women

Our study's findings shed light on the characteristics of Omani women who have preeclampsia (PE). Women with PE had considerably higher systolic and diastolic blood pressure as well as a higher prevalence of proteinuria (*p* < 0.001 for all), as predicted by the diagnostic criteria. This is in line with the fundamental diagnostic criteria for the illness and validates our case-control classification (1, 4).

Beyond these definitional characteristics, shorter gestational age at birth and higher gravidity were found to be significant factors independently associated with PE in our multivariate analysis, which did not include blood pressure or proteinuria. This is consistent with the knowledge that shorter gestation is an important clinical signal rather than just a result because the pathophysiology of PE frequently requires an earlier birth (37). Other regional investigations have revealed the connection with increased gravidity, which contrasts with the traditional primigravida risk profile (21, 38). This could be due to a different underlying risk architecture in multiparous women, which could be linked to comorbidities from many pregnancies, accumulating endothelial susceptibility, or increasing maternal age. The observed association between higher gravidity and PE in this cohort differs from the traditionally recognized primigravida risk profile. One possible explanation is the influence of increasing maternal age with successive pregnancies, although maternal age itself was not independently significant in the adjusted model. Additionally, repeated pregnancies may contribute to cumulative endothelial dysfunction, metabolic alterations, or chronic inflammatory changes that increase susceptibility to hypertensive disorders. Population-specific reproductive characteristics, including shorter interpregnancy intervals and high parity patterns in the Gulf region, may also contribute to this association. Similar findings have been reported in selected studies from low- and middle-income settings, suggesting that PE risk profiles may vary across populations. Clinically, this finding highlights the importance of considering reproductive history and gravidity during antenatal risk stratification rather than focusing exclusively on primigravida status.

Despite elevated BMI being a globally recognized risk, preeclamptic women in our study had a considerably higher mean BMI (*p* < 0.001). Although women with PE demonstrated a significantly high mean BMI in univariate analysis, BMI did not remain an independent predictor in the adjusted multivariable model. Nevertheless, obesity remains an important clinically recognized risk factor for PE and should continue to be considered during antenatal risk assessment for early identification of women at increased risk of preeclampsia. Preconception weight control is crucial as a preventive measure because obesity leads to endothelial dysfunction and a pro-inflammatory state, both of which are essential to the pathophysiology of PE (39). Notably, several other covariates, such as maternal age, parity, history of GDM, hemoglobin, and platelet count, did not show any significant independent association in our logistic regression model. This implies that the timing of clinical presentation (gestational age) and reproductive history (gravidity) are the main non-definitional risk markers in this Omani cohort. These results highlight the significance of a thorough risk assessment that goes beyond standard laboratory testing and vital signs.

## Study strengths and limitations

This study presents the first exploration of the prevalence of PE and associated non-definitional risk factors in Oman. The inclusion of both preeclamptic and non-preeclamptic women from national referral hospitals (2021–2023) resulted in a large, diverse study population. High-quality health information systems and medical birth records ensured robust data collection, with independent execution minimizing biases. As the first study on PE in Oman, the study provided a valuable reference and attempted to bridge the knowledge gap. The use of a case-control design helped to reduce selection bias and enhance generalizability. The documentation of risk factors for PE and other potential risks, including gravidity and BMI, provides important substrates for future research and targeted interventions to improve outcomes, especially for women and their newborns.

The study also has limitations, such as the use of a retrospective design that relies on pre-existing data, which may contain errors and missing data since it is not primarily corrected with research intentions. The retrospective nature also hinders establishing an accurate chronological sequence of events, especially concerning risk factors. Additionally, because the study was conducted in tertiary referral hospitals using a retrospective case-control design, the reported proportion of PE reflects a hospital-based cohort estimate and should not be interpreted as a national population prevalence. Referral bias may have contributed to a higher proportion of high-risk pregnancies within the study cohort. Furthermore, causal relationships between associated factors and PE cannot be definitively established due to the observational retrospective design. Data extraction from electronic medical records omitted variables like patients’ education level and occupation, which might influence health literacy, health behaviors and practices, and PE awareness. Future studies may benefit from a prospective design for comprehensive data collection and accuracy.

## Implications for practice

These results necessitate practical adjustments to prenatal treatment in Oman. To identify high-risk women early, clinicians should use pre-pregnancy BMI and parity in routine risk stratification. In primary care, preconception weight management programs are an essential preventive strategy. Additionally, healthcare facilities could adopt standardized hypertension monitoring procedures and referral mechanisms to improve early detection. To empower women and encourage prompt care-seeking, community-based education on preeclampsia symptoms must be enhanced concurrently. When combined, these actions can dramatically lower the morbidity associated with preeclampsia.

## Conclusion

This study offers the first quantitative evaluation of preeclampsia in Omani women, reporting a cohort prevalence of 25.0%. Higher gravidity and shorter gestational age were found to be significant independent factors. These results demonstrate the importance of non-definitional elements in risk assessment for this population. The findings highlight the significance of preconception health measures and the need to incorporate parity and pregnancy history into early antenatal risk assessment. The study creates a critical national baseline and provides useful information to direct clinical practice and public health policy aimed at mitigating the burden of preeclampsia in Oman.

## Data Availability

The raw data supporting the conclusions of this article will be made available by the authors, without undue reservation.
